# Radiographic Evaluation of Meniscal Extrusion

**DOI:** 10.7759/cureus.3262

**Published:** 2018-09-06

**Authors:** Behrad Golshani, Sara Bamrungchart, Cyrus P Bateni

**Affiliations:** 1 Radiology, David Geffen School of Medicine at University of California Los Angeles (UCLA), Los Angeles, USA; 2 Radiology, University of California Davis School of Medicine, Bangkok, THA; 3 Radiology, University of California Davis Schoo, Sacramento, USA

**Keywords:** meniscus, knee, osteoarthritis, radiograph, extrusion, extrusion

## Abstract

Background

Magnetic resonance imaging (MRI) is well established as the preferred noninvasive tool for meniscal evaluation. To our knowledge, there has been no study examining the utility of diagnosing meniscal extrusion from radiography alone. We hypothesize that with appropriate window settings, meniscal extrusion may be diagnosed on radiography with high sensitivity and specificity.

Materials and methods

We included 190 patients with MRI of the knee performed within three months of knee radiography. As defined within the literature, we utilized the MRI criteria of meniscal extrusion as meniscal tissue extending 3 mm or greater beyond the tibial plateau, excluding osteophytes. Two attending radiologists blindly and independently identified the absence or presence, in millimeters, of medial meniscal extrusion on plain film radiography. Kappa test and Pearson correlation coefficient were calculated to assess the extent of inter-reader agreement and correlation. Sensitivity and specificity were calculated for each reader, assuming the concurrent MRI served as the gold standard.

Results

Ninety-six patients had medial meniscal extrusion and 94 had no medial extrusion by MRI. Kappa test for inter-reader agreement = 0.61. Pearson coefficient for inter-reader measurement correlation = 0.69. Reader A had sensitivity of 0.59 (95% CI 0.49-0.69) and specificity of 0.88 (95% CI 0.79-0.94). Reader B had sensitivity of 0.61 (95% CI 0.51-0.71) and specificity of 0.85 (95% CI 0.76-0.91).

Conclusion

There is substantial inter-reader agreement and high correlation of meniscal extrusion measurement between readers. Our results suggest that while radiographs have low sensitivity for evaluation of meniscal extrusion, their high specificity may be of clinical utility.

## Introduction

The meniscus of the knee is a fibrocartilagenous structure that plays a long-established, significant role in maintaining the complex biomechanics of the knee joint [1]. Injury to the meniscus is exceedingly common and the loss of appropriate meniscal function is known to predispose and advance osteoarthritis [2]. In turn, osteoarthritis is the most common cause of musculoskeletal disability in the United States [3]. More specifically, meniscal tears and extrusions have been identified as risk factors for progression of osteoarthritis [4]. Meniscal extrusion has traditionally been defined as meniscal tissue extending 3 mm or greater beyond the edge of the tibial plateau, excluding marginal osteophytes, measured on magnetic resonance imaging (MRI) [5]. More recent literature has suggested that decreasing the threshold of extrusion to 2 mm may also be significant [6]. Much research has been devoted to the association of meniscal extrusion with tears and subsequent degenerative change. However, literature regarding radiographic changes associated with meniscal pathology is sparse. We hypothesized that with the proper window settings, meniscal extrusion can be confidently detected on standard knee radiographs.

## Materials and methods

Using a keyword search of MRI reports with the search term “meniscal extrusion,” we collected 200 MRIs of patients who had undergone both a knee MRI and knee radiographs within a three-month timeframe from December 2013 to March 2015. Meniscal extrusion on MRI was defined as meniscal tissue extending 3 mm or greater beyond the medial margin of the medial tibial plateau, excluding osteophytes. Measurement on MRI was performed by an independent reader in the coronal plane at the level of the mid anteroposterior tibial plateau on a proton density weighted, fat-saturated sequence with a relaxation time of 3200 ms, echo time of 47 ms, field of view of 14 x 14 cm, slice thickness of 3.5 mm with a gap of 0.5 cm, and matrix of 384 x 256.

Two musculoskeletal fellowship-trained attending radiologists with three and five years experience participated in a small workshop demonstrating appropriate windowing of radiographs for detection of meniscal tissue at a standard diagnostic radiology workstation.

**Figure 1:**
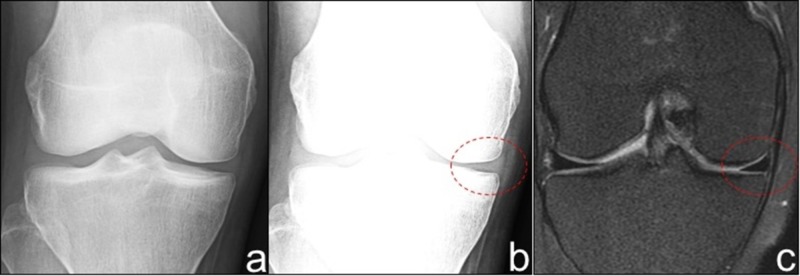
Radiographic appearance of a normally positioned meniscus. a) AP knee radiograph with standard window settings. b) AP knee radiograph with narrow window settings demonstrates visualized meniscal tissue without extrusion. c) Correlative mid coronal proton density fat saturation MRI confirms lack of meniscal extrusion. MRI - magnetic resonance imaging.

**Figure 2:**
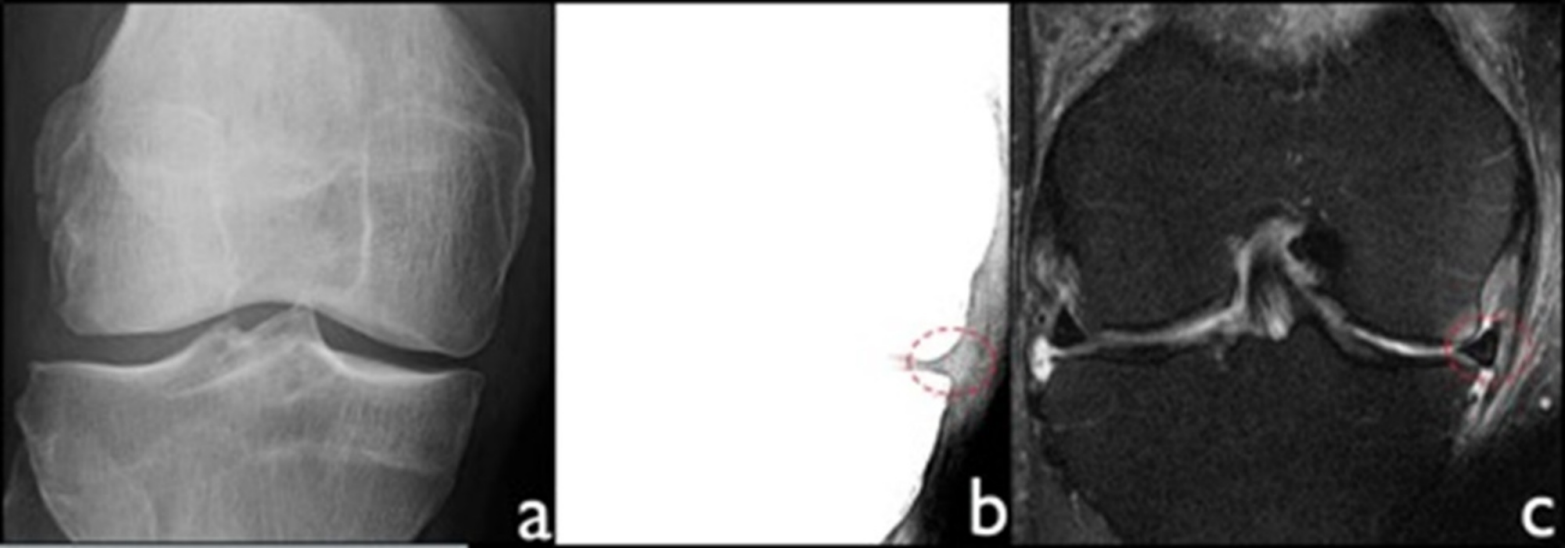
Radiographic appearance of meniscal extrusion. a) AP knee radiograph with standard settings. b) AP knee radiograph with narrow window settings demonstrates medial meniscal extrusion greater than 3 mm as measured by tissue extending beyond the medial tibial plateau, excluding osteophytes. c) Correlative coronal proton density fat saturation MRI confirms presence of extrusion. MRI - magnetic resonance imaging.

Radiographic examples of medial meniscal extrusion and no meniscal extrusion were demonstrated (Figures 1-2) as was the appropriate windowing for detection of meniscal extrusion on a total of 10 cases. The two readers then blindly and independently identified either the absence or the presence, to the nearest millimeter, of medial meniscal extrusion on an anteroposterior knee radiograph.

McNemar’s test and simple Kappa were performed using the Statistical Analysis System (SAS) software (SAS Institute Inc., NC, USA) to assess the extent of agreement between the two readers and the MRI. Sensitivity and specificity were calculated based on the contingency table for each reader assuming the MRI as the gold standard.

Our institutional review board approved this retrospective health insurance portability and accountability act (HIPAA) compliant study.

## Results

A total of 200 patients were evaluated for inclusion in this study with an intended equal split of patients with and without extrusion by MRI. Of these patients, 10 were excluded based on age less than 18 and inadequate radiograph due to suboptimal patient positioning. Subsequently, 190 patients were included and the readers were blind to the intended ratio of patients with and without extrusion. As determined by MRI, 96 patients had medial meniscal extrusion (mean age of 58.8 years; 61 females, 35 males) and 94 had no medial extrusion by MRI (mean age of 46.4 years; 60 females, 34 males). For the detection of meniscal extrusion on radiograph as compared to MRI, Reader A (Table 1) had a sensitivity of 0.59 (95% CI 0.49-0.69) and a specificity of 0.88 (95% CI 0.79-0.94); Reader B (Table 2) had a sensitivity of 0.61 (95% CI 0.51-0.71) and a specificity of 0.85 (95% CI 0.76-0.91). Kappa test for inter-reader agreement = 0.61. Pearson coefficient for inter-reader measurement correlation = 0.69.

**Table 1:** Reader A interpretation of extrusion on radiograph versus MRI. MRI - magnetic resonance imaging.

Reader A		MRI		Totals		Sensitivity = 0.59	Specificity = 0.88
		Extrusion	No extrusion				
Radiograph	Extrusion	57	11	68			
	No Extrusion	39	83	122			
Totals		96	94				

**Table 2:** Reader B interpretation of extrusion on radiograph versus MRI. MRI - magnetic resonance imaging.

Reader B		MRI		Totals		Sensitivity = 0.61	Specificity = 0.85
		Extrusion	No extrusion				
Radiograph	Extrusion	59	14	73			
	No Extrusion	37	80	117			
Totals		96	94				

## Discussion

Meniscal extrusion is a recognized imaging finding that has been associated with a posterior root tear of the meniscus and osteoarthritis [7]. When associated with root tears, medial meniscal extrusion has been reported to be three times more prevalent as compared to lateral meniscal extrusion [8]. Further, medial meniscal extrusion has also been linked to medial collateral ligament (MCL) edema on MRI [9]. The resulting alteration of biomechanics secondary to meniscal extrusion may then precipitate cartilage damage and subsequent osteoarthritic changes [10]. Despite radiography being the most appropriate initial examination of both traumatic and nontraumatic knee pain per the American College of Radiology (ACR) appropriateness criteria [11, 12], research on the radiographic evaluation of menisci is more sparse than the assessment by MRI. Specifically, our study is the first to evaluate the detection of medial meniscal extrusion on radiographs.

Our results show that with appropriate window settings and limited, targeted training, identification of meniscal extrusion can be performed with high specificity. A positive result, therefore, is unlikely to represent a false positive and could render a plausible cause for the etiology of a patient’s symptoms. Given that the imaging evaluation of knee pain is most appropriately initiated with radiography, a positive finding of meniscal extrusion could determine the need for proper clinical consultation and the potential utility of additional imaging such as MRI. Further, the results demonstrate substantial inter-reader agreement, k=0.61 [13], suggesting that this may be feasible in routine clinical practice. We would propose that, when appropriate, meniscal evaluation could be incorporated into a radiologist’s search pattern with ease and accuracy. Expectedly, there was low sensitivity for extrusion on radiography by both readers, as regardless of the modification of window settings, radiography does not allow for the same degree of soft tissue contrast as MRI. A negative result for extrusion by radiograph is consequently not reliable nor as clinically useful as a positive result.

Limitations of the study are lack of correlation with arthroscopy, as the majority of the patients in our study did not undergo subsequent arthroscopy. We aimed to correlate the radiographic appearance of meniscal extrusion with that of MRI, and the actual presence of meniscal pathology by arthroscopy was outside the scope of the study and not accounted for. Our study was also limited to the medial meniscus - the lateral meniscus was not evaluated due to the relative infrequency of the occurrence of lateral meniscal extrusion based upon preceding search criteria. While it is uncertain if these results can be transferred to the radiographic evaluation of lateral meniscal extrusion, other studies have shown that radiographs can be utilized for lateral meniscal evaluation and this may warrant further evaluation [14]. Our search criteria also yielded both weight-bearing and nonweight-bearing radiographs, which were not analyzed separately. Given that meniscal extrusion would theoretically be more pronounced on weight-bearing radiographs, this may have in part accounted for our low sensitivity.

In summary, while presence of meniscal extrusion by radiography does not necessarily obviate the need for further imaging, it may provide a quick, reliable adjunct to routine evaluation of knee radiographs. A positive finding could bring closer attention to the potential etiology of the patient's symptoms and direct management of this particular subgroup.

## Conclusions

Meniscal extrusion is known to be associated with meniscal tears, which lead to loss of meniscal hoop strength and subsequent development of osteoarthritis. MRI has been known to have high accuracy for identifying meniscal pathology. Though radiographs are often the first line of imaging for patients with knee pain, there has been little to no reported literature on the use of radiography to evaluate the meniscus. Our results demonstrate that with brief, targeted training, the presence of meniscal extrusion can be identified radiographically with high specificity. These results may support the utility of training radiologists to evaluate meniscal extrusion on routine radiographs performed for knee pain.
